# The RNA-binding protein RBP42 regulates cellular energy metabolism in mammalian-infective *Trypanosoma brucei*

**DOI:** 10.1128/msphere.00273-23

**Published:** 2023-08-15

**Authors:** Anish Das, Tong Liu, Hong Li, Seema Husain

**Affiliations:** 1 Department of Microbiology, Biochemistry and Molecular Genetics, Rutgers-New Jersey Medical School, Newark, New Jersey, USA; 2 Center for Advanced Proteomics Research, Rutgers-New Jersey Medical School, Newark, New Jersey, USA; 3 Genomics Center, Rutgers-New Jersey Medical School, Newark, New Jersey, USA; University of Texas Southwestern, Dallas, Texas, USA

**Keywords:** RNA-binding protein, RBP42, iCLIP, RNA regulon, *Trypanosoma*, protozoa, metabolism

## Abstract

RNA-binding proteins (RBPs) are key players in coordinated post-transcriptional regulation of functionally related genes, defined as RNA regulons. RNA regulons play particularly critical roles in parasitic trypanosomes, which exhibit unregulated co-transcription of long unrelated gene arrays. In this report, we present a systematic analysis of an essential RBP, RBP42, in the mammalian-infective bloodstream form of African trypanosome and show that RBP42 is a key regulator of parasite’s central carbon and energy metabolism. Using individual-nucleotide resolution UV cross-linking and immunoprecipitation to identify genome-wide RBP42-RNA interactions, we show that RBP42 preferentially binds within the coding region of mRNAs encoding core metabolic enzymes. Global quantitative transcriptomic and proteomic analyses reveal that loss of RBP42 reduces the abundance of target mRNA-encoded proteins, but not target mRNA, suggesting a positive translational regulatory role of RBP42. Significant changes in central carbon metabolic intermediates, following loss of RBP42, further support its critical role in cellular energy metabolism.

*Trypanosoma brucei* infection, transmitted through the bite of blood-feeding tsetse flies, causes deadly diseases in humans and livestock. This disease, if left untreated, is almost always fatal. Existing therapies are toxic and difficult to administer. During *T. brucei’s* lifecycle in two different host environments, the parasite progresses through distinctive life stages with major morphological and metabolic changes, requiring precise alteration of parasite gene expression program. In the absence of regulated transcription, post-transcriptional processes mediated by RNA-binding proteins play critical roles in *T. brucei* gene regulation. In this study, we show that the RNA-binding protein RBP42 plays crucial roles in cellular energy metabolic regulation of this important human pathogen. Metabolic dysregulation observed in RBP42 knockdown cells offers a breadth of potential interest to researchers studying parasite biology and can also impact research in general eukaryotic biology.

## INTRODUCTION

RNA regulons that coordinately regulate the production of functionally related proteins are emerging as key regulatory modules in eukaryotes (1). In contrast to the prokaryotic “operons” in which functionally related gene clusters are co-transcribed and co-translated, RNA regulons co-regulate gene cohorts post-transcriptionally by dynamic RNA-protein and RNA-RNA interactions to modulate critical regulatory steps, including messenger RNA (mRNA) maturation, localization, translation, and decay (2). Thus, RNA regulons allow rapid, yet precise response to both intra- and extra-cellular signals that trigger readjustment of entire biochemical pathways (3). Accordingly, RNA regulons have been discovered in diverse cell systems performing crucial regulatory functions (4, 5).

RNA regulon-mediated post-transcriptional regulation is particularly important in *Trypanosoma brucei* subspecies, a deadly group of parasitic protozoa that cause human African trypanosomiasis (HAT) and the cattle disease Nagana (6 – 8). *T. brucei*, with a complex life cycle in two very different host environments, mammals and tsetse flies, undergo major morphological and metabolic changes that require precise coordination of specific gene expression patterns (9). However, the profoundly unusual gene arrangement in *T. brucei*, where almost all protein-coding genes are co-transcribed as long polygenic arrays, prohibits the typical transcriptional regulation of individual genes (10, 11). Instead, regulation is primarily achieved at the post-transcriptional level (12). Thus, RNA-binding proteins (RBPs), by interacting with specific sets of mRNAs, play key roles in controlling gene cohorts that are essential to maintain, or drive, developmental progression (7, 13, 14). Recent studies have established the critical roles of RBPs in trypanosome biology; overexpression of single RBP can force lifecycle-specific changes in the parasite (15, 16).

*T. brucei* central carbon and energy metabolism is a highly regulated process and undergoes striking changes in different host environments, dictated primarily by the availability of nutrients (17, 18). For example, in the tsetse fly midgut, the procyclic form harbors full repertoire of glycolytic enzymes as well as mitochondrial Krebs cycle and electron transport chain (19). Although in glucose-rich culture media the procyclic forms preferentially metabolize glucose through glycolysis, in the fly midgut, abundant amino acid, such as proline, is metabolized in the mitochondria and ATP is produced mainly by oxidative phosphorylation via a canonical cytochrome-containing electron transport chain (20). In contrast, in mammalian blood, the slender bloodstream form uses glycolysis as the sole source of ATP (21, 22). Existing evidence suggests that slender bloodstream forms have no detectable Krebs cycle or mitochondrial respiratory chain coupled to ATP synthesis. Similar metabolic adjustments are also observed in trypanosomes growing in other varying environmental niches (23, 24). Although the molecular mechanisms that enable these dramatic metabolic remodeling are not yet fully understood, studies suggest critical roles performed by RBPs in this regulation (25 – 28).

*T. brucei* RNA-binding protein RBP42 shares sequence homology to metazoan RAS-GTPase activating protein SH3 domain binding protein (G3BP) (29 – 31). Metazoan G3BP proteins are a family of mRNA-binding proteins that are known to regulate gene expression in response to intra- and extra-cellular stimuli. Multiple modes of function are attributed to G3BP proteins, which include mRNA stabilization and destabilization, subcellular localization, and translation (32, 33). RBP42 is known to interact with translating, polysome-associated mRNAs encoding enzymatic proteins of central carbon metabolic pathways in procyclic trypanosomes (29). However, the significance of these interactions in trypanosome gene regulation remains unknown.

In this report, we establish RBP42 as a post-transcriptional regulator of trypanosome central carbon and energy metabolism. Using protein-RNA crosslinking via ultraviolet (UV)-irradiation, we captured *in vivo* RBP42-RNA interactions in mammalian-infective slender bloodstream form *T. brucei* (34). Identification of RBP42 crosslink sites on target transcripts shows that RBP42 binds within the coding sequence of many mRNAs that encode enzymatic proteins involved in cellular core metabolic pathways. Applying a conditional knockdown system, we show that RBP42 does not affect target mRNA stability; loss of RBP42 has minimal effect on target mRNA abundance. Instead, RBP42 promotes target mRNA translation; loss of RBP42 markedly reduces target mRNA-encoded protein abundance. Importantly, loss of RBP42 causes drastic reduction of many metabolic intermediates of the central carbon metabolic process, as well as ATP, NAD, and NADP that are critical for cellular energy and redox homeostasis. These data indicate that RBP42 plays an essential role in fine-tuning trypanosome energy metabolism, whose regulation is essential for *T. brucei*’s parasitic lifestyle.

## RESULTS

### RBP42 binds within the coding region of transcripts encoding cellular primary metabolic enzymes

**Fig 1:**
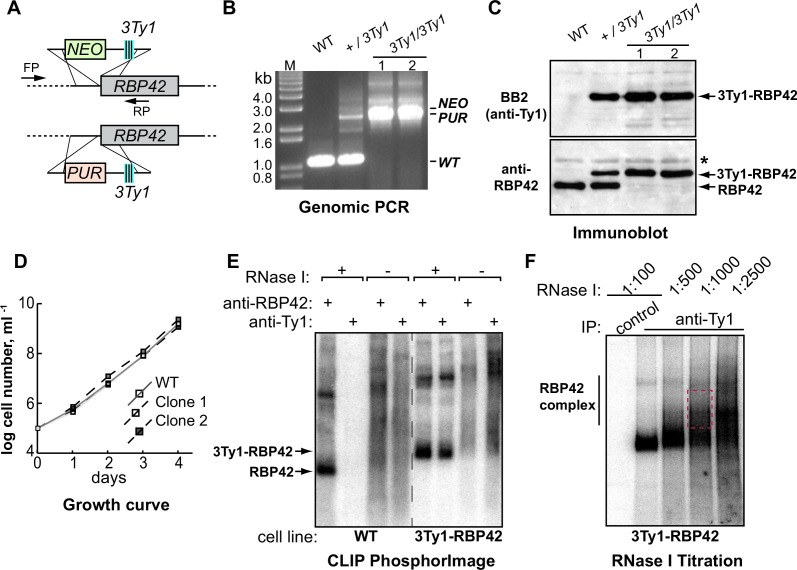
Crosslink immunoprecipitation of RBP42-RNA complex from slender bloodstream form *T. brucei*. (**A**) Schematic showing homologous recombination-mediated *in situ* tagging to generate homozygous 3Ty1-epitope tagged RBP42 cell line. PCR generated DNA cassettes, containing neomycin (*NEO*) and puromycin (*PUR*) selectable marker genes, are shown. (**B**) Analysis of genomic DNA by PCR using FP and RP primers shown in panel (**A**) verifies proper integration of the DNA cassettes in two representative clonal cell lines. (**C**) Immunoblot analysis of total cellular proteins confirms the presence of only 3Ty1-tagged RBP42 protein in these clonal transgenic cell lines. Whereas anti-RBP42 antibody detects both native RBP42 and 3Ty1-tagged RBP42 proteins, anti-Ty1 (BB2) antibody detects only the tagged protein. Asterisk marks non-specific binding of anti-RBP42 antibody to an unknown protein. (**D**) Growth analysis shows that both clonal cell lines grow comparably to wild-type (WT) cells. (**E**) Immunoprecipitated protein-RNA complexes that were 5′-end radiolabeled on their RNA. Complexes were separated by denaturing sodium dodecyl sulfate polyacrylamide gel electrophoresis (SDS-PAGE) and transferred to nitrocellulose membranes. A PhosphorImage scan of ^32^P-labeled protein-RNA complexes purified from both wild-type (WT) cells and 3Ty1-RBP42 cells is shown. In the absence of ribonuclease (RNase I), the protein-RNA complexes are too large, which fail to resolve by gel chromatography, and appear as broad smear. While anti-Ty1 (BB2) antibody immunoprecipitated only the tagged protein, anti-RBP42 antibody immunoprecipitated both WT and tagged proteins. (**F**) Optimization of RNase I concentration to obtain optimal size RNA fragments for iCLIP library preparation. A representative PhosphorImager scan, similar to panel **E**, is shown. Protein-RNA complexes were treated with decreasing concentrations of RNase I before immunoprecipitation. Complexes from the marked gel area (red dotted box) were excised from the nitrocellulose membrane, and extracted RNAs were reverse transcribed to prepare cDNA library. Normal mouse antibody serves as control.

To accurately identify RBP42’s targets in mammalian-infective slender bloodstream form *T. brucei*, we performed UV cross-linking and immunoprecipitation (CLIP) by incorporating two strategies. First, to eliminate non-specific protein-antibody interactions from our analysis, we used a second independent antibody reagent, a mouse monoclonal antibody to Ty1-epitope (anti-Ty1), in addition to a rabbit polyclonal antibody to bacteria-derived recombinant RBP42 protein (anti-RBP42) (29). Transgenic cell line (3Ty1-RBP42), producing triple-Ty1 epitope-tagged RBP42 protein, was generated by homologous recombination-mediated *in situ* tagging of both alleles of the diploid parasite (Fig. 1A). Genomic polymerase chain reaction (PCR) and immunoblot analyses of two representative clonal cell lines show proper integration and expression of the tagged-RBP42 protein (Fig. 1B and C). 3Ty1-RBP42 cells that solely depend on tagged-RBP42 protein grow comparably to wild-type (WT) cells, demonstrating proper function of the tagged protein (Fig. 1D). Both antibodies, anti-Ty1 and anti-RBP42, efficiently immunopurified crosslinked RBP42-RNA complexes from UV-irradiated 3Ty1-RBP42 cellular extracts. However, only anti-RBP42 efficiently immunopurified RBP42-RNA complexes from UV-irradiated WT cellular extracts, as expected (Fig. 1E). We used WT cells in combination with anti-RBP42 antibody and 3Ty1-RBP42 cells in combination with anti-Ty1 antibody, to identify RBP42’s cellular RNA targets (Fig. 1E and F). Second, since UV-irradiated cross-linking of protein-RNA interactions is known to be very inefficient (35, 36), we captured RBP42-RNA interactions at two increasing UV doses, at 150 mJ/cm^2^ and at 300 mJ/cm^2^ constant energy. We reasoned that authentic RBP42-RNA interactions, as opposed to non-specific, cryptic interactions, would be captured at both conditions.

**Fig 2:**
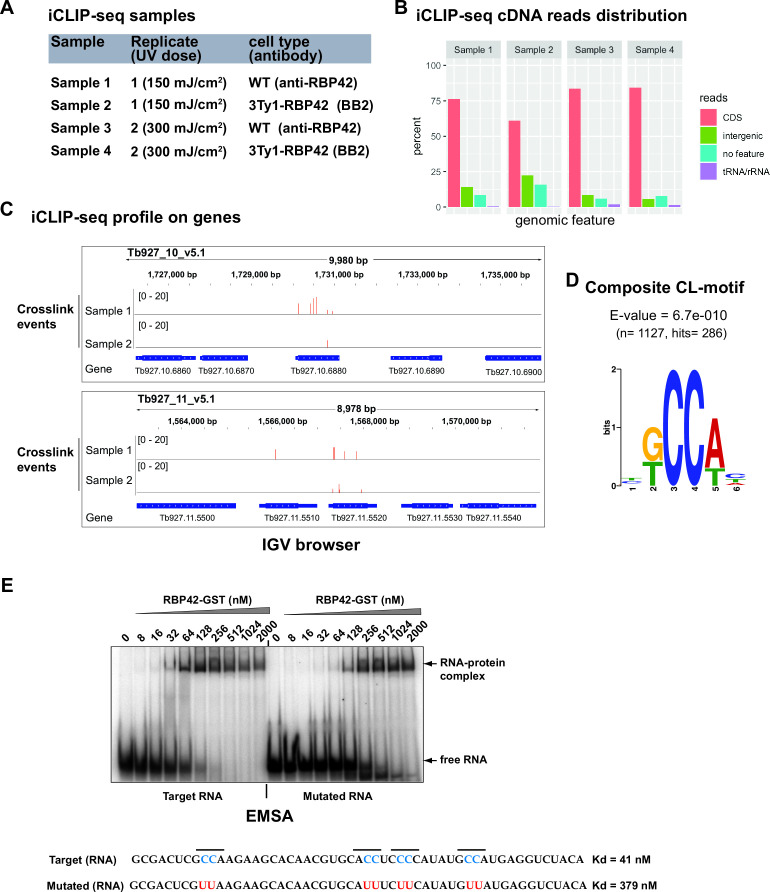
iCLIP-seq identifies RBP42’s RNA targets in slender bloodstream form *T. brucei*. (**A**) Four iCLIP-seq cDNA libraries (Samples 1–4) were made from both wild-type (WT) cells, using anti-RBP42 antibody (Samples 1 and 3), and 3Ty1-RBP42 cells using anti-Ty1 (BB2) antibody (Samples 2 and 4), applying two increasing UV doses, 150 mJ/cm^2^ (Samples 1 and 2) and 300 mJ/cm^2^ (Samples 3 and 4). (**B**) RBP42 predominantly binds within the coding region of mRNA targets. Bar plots of cDNA read distributions, mapped to *T. brucei* genomic features (*T. brucei brucei* strain 927 genome from TriTrypDB.org—version 46), are shown. CDS and intergenic denote the protein-coding open reading frames and the 5′ and 3′ untranslated regions of mRNA, respectively. tRNA/rRNAs are as defined in the database mentioned above, and no features are without any annotation. (**C**) IGV browser view of RBP42 crosslinking to two candidate mRNA targets, one on chromosome 10 (Tb927.10.6880—glyceraldehyde 3-phosphate dehydrogenase, cytosolic) and the other on chromosome 11 (Tb927.11.5520—triosephosphate isomerase). A crosslink event is counted for each unique cDNA and assigned to the upstream crosslink nucleotide (see Materials and Methods). The red bars indicate the number of crosslink events on crosslink sites. Few neighboring non-target genes are also shown as internal controls to indicate specificity of RBP42 interactions. (**D**) The composite sequence motif associated with RBP42 is discovered by comparing significant crosslink clusters using DREME algorithm (meme-suite.org). (**E**) PhosphorImage analysis of EMSA shows complexes of RBP42 protein with target and mutated RNA substrates. RNA sequences and dissociation constants (Kd) are indicated.

We identified RBP42-RNA interactions by generating and deep-sequencing cDNA libraries from CLIP-purified RNA (Fig. 1F). cDNA libraries were made using individual-nucleotide resolution CLIP (iCLIP) method (37). The iCLIP, by incorporating a cDNA circularization step, takes advantage of the frequent termination of reverse transcriptase (RT) at the crosslink site, which therefore corresponds to the nucleotide preceding the cDNA start. Thus, iCLIP enables the position of RBP-RNA interaction to be precisely mapped, and each unique cDNA molecule in the library denotes an individual crosslink event. We produced four independent iCLIP libraries: two for each of anti-RBP42 antibody (Samples 1 and 3) and anti-Ty1 antibody (Samples 2 and 4) at both 150 mJ/cm^2^ (Samples 1 and 2) and 300 mJ/cm^2^ UV doses (Samples 3 and 4) (Fig. 2A). We obtained 29,464 and 26,055 unique cDNA molecules in Samples 1 and 2, respectively, and 867,214 and 493,361 unique cDNA molecules in Samples 3 and 4, respectively (see Fig. S1a at https://doi.org/10.6084/m9.figshare.21737321). The large, ~20-fold more unique cDNA molecules in Samples 3 and 4, compared to Samples 1 and 2, may occur as a batch effect, i.e., minor variations in enzymatic activities and/or reaction conditions. It is also possible that higher, 300 mJ/cm^2^, UV doses in Samples 3 and 4 produced more crosslink events (see Fig. S1b at https://doi.org/10.6084/m9.figshare.21737321). Genome-wide comparison of mapped crosslink sites reveals moderate to high degree of correlations (Spearman correlations from 0.40 to 0.94 between different samples) among all four iCLIP libraries (see Fig. S1c at https://doi.org/10.6084/m9.figshare.21737321). Distribution of crosslink sites over annotated genomic features show that the vast majority of RBP42 interactions occur within the mRNA coding sequences (Fig. 2B). This result, in accordance with previously published data in fly-infective procyclic forms (29), confirms that RBP42 predominantly binds within the target mRNA-coding sequences.

We identified RBP42-target transcripts by recording crosslink sites to annotated *T. brucei* 927 genome (TriTrypDB.org; V45). The number of crosslink events at each recorded crosslink site was calculated by counting the number of stacked unique cDNAs with coinciding start sites (see Materials and Methods) (Fig. 2C and see Fig. S2 at https://doi.org/10.6084/m9.figshare.21737321). Evaluation by PureCLIP algorithm (38), designed to analyze iCLIP data, identified similar crosslink sites. To identify potential RBP42 crosslink sequence motif, we sampled ~1,100 high-confidence crosslink site sequences, each 11 nucleotides (nt) long, consisting of crosslink site plus 5 nt flanking sequences, and searched for motif discovery using MEME suite (https://meme-suite.org/tools/meme). A consensus hexanucleotide sequence, centered around a CC dinucleotide, with highly significant enrichment (*E* = 6.7e−010) is revealed as the preferential RBP42 crosslink sequence motif (Fig. 2D). Since RBP42 harbors single RNA recognition motif (RRM) domain, the shortness of the sequence motif is consistent with RNA recognition by other RRM domain-containing proteins (39). *In vitro* binding assays show specific binding of RBP42 to a candidate target sequence (see Fig. S3 at https://doi.org/10.6084/m9.figshare.21737321). Substitution of the CC dinucleotides within the target sequence decreased but did not abolish the binding affinity (Fig. 2E).

**Fig 3:**
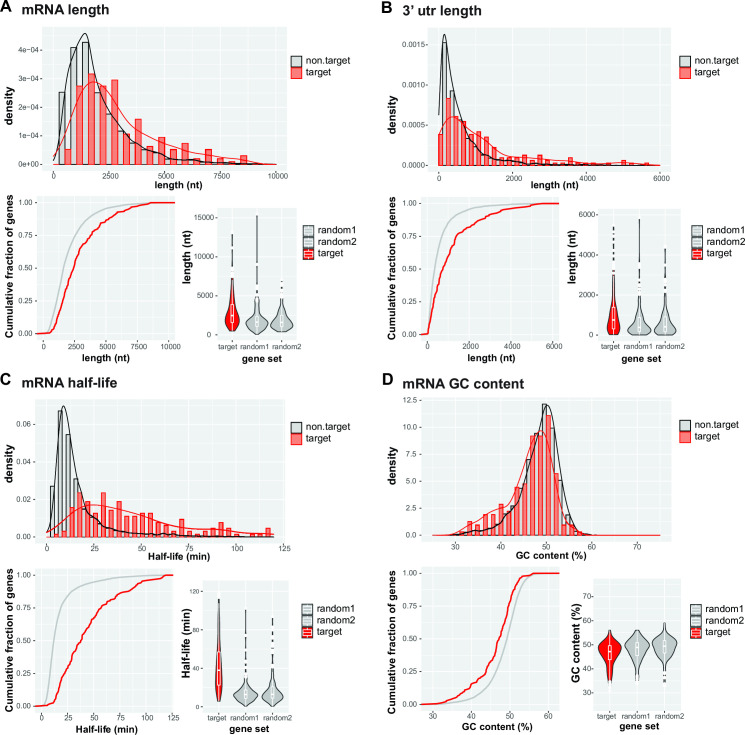
RBP42-targets represent a set of relatively long, stable mRNAs. Panels **A** and **B** show the density and the cumulative distribution frequency plots of mRNA lengths, and 3′UTR lengths of 183 RBP42-target mRNAs (red) compared to non-target background sets (gray). Violin plots show the distributions of the 183 RBP42-target mRNAs and 3′UTR lengths (target, red), compared to two control mRNA sets of equal size, randomly sampled from the database (random1 and random2, gray). Panels **C** and **D** show similar plots for *T. brucei* bloodstream form mRNA half-lives and GC content.

Tabulation of RBP42 targets, compiled from all four samples, resulted a combined and overlapping set of 2,145 transcripts: 796 in Sample 1, 903 in Sample 2, 1,449 in Sample 3, and 1,020 in Sample 4 (see Fig. S1d at https://doi.org/10.6084/m9.figshare.21737321). To undertake the functional significance of RBP42 binding on target transcripts, we classified the “most reliable” RBP42-target set using a stringent criterion—189 congruent transcripts identified in all four iCLIP libraries. The “most reliable” RBP42-target mRNAs represent a set of relatively stable (median half-life of 40 min compared to 12 min for the non-target set) (40), long mRNAs (median length of 2.5 kb compared to 1.6 kb for the non-target set) with long 3′ untranslated region (UTR) (median 3′UTR length of 800 nt compared to 360 nt for the non-target set) (Fig. 3). We validated RBP42’s *in vivo* association by performing independent CLIP experiments in which we measured the enrichment of a representative set of target transcripts using anti-RBP42 antibodies relative to non-specific antibody control. Indeed, all 10 target transcripts were enriched, from 2.5- to 7.5-fold, but 3 non-target transcripts were not (see Fig. S4a at https://doi.org/10.6084/m9.figshare.21737321). The 189 transcripts encode 181 ortholog protein groups that include 16 of unknown function and therefore annotated as hypothetical protein. Gene ontology (GO) analysis of 165 known protein groups shows several enriched GO terms associated with primary metabolic processes (see Fig. S4b at https://doi.org/10.6084/m9.figshare.21737321). Similar result was reported with RBP42-targets in fly-infective procyclic forms (29), indicating a conserved RBP42 function in both procyclic and slender bloodstream stages of the parasite. We also observed that RBP42-targets include transcripts encoding several RNA binding proteins, 18 in the “most reliable” 189-target set including its own, and several translation factors, indicating a critical RBP42 function in modulating parasite’s RBP-mediated post-transcriptional regulation. Interestingly, many tRNAs, five tRNAs in the “most reliable” set, were crosslinked to RBP42 (see Table S1 at https://doi.org/10.6084/m9.figshare.21737321). Since RBP42 is known to be associated with translating polysomes (29), crosslinking to tRNAs is not surprising. However, the exact mechanism of how RBP42 connects both mRNA and tRNA within the translating polysomes is not known.

### RBP42 is essential for slender bloodstream form *T. brucei* survival

**Fig 4:**
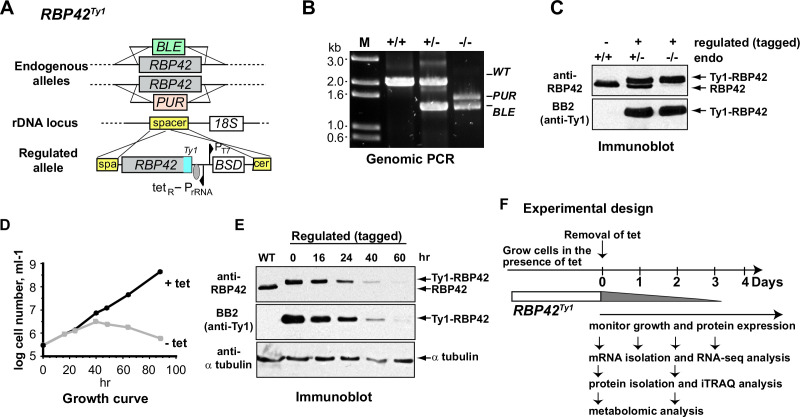
RBP42 is essential for slender bloodstream form *T. brucei*. (**A**) Schematic shows the strategy used to generate RBP42 conditional knockdown cell line (RBP42^Ty1^). Homologous recombination-mediated replacement by two antibiotic resistance genes inactivated two endogenous alleles. Cellular RBP42 expression is maintained from an exogenous tetracycline-inducible Ty1-tagged RBP42 allele inserted in an rRNA spacer region. (**B**) Analysis of genomic DNA by PCR, as in Fig. 1, verifies proper integration of the antibiotic resistance DNA cassettes. (**C**) Immunoblot confirms expression of only the Ty1-tagged RBP42 protein from the regulated allele in RBP42^Ty1^ cells. Antibodies are described in Fig. 1. (**D**) Growth analysis shows that RBP42 is essential for cell viability. RBP42^Ty1^ cells were maintained by adding tetracycline to the growth media. RBP42 expression was turned off by removing tetracycline from the growth media. Parasites were counted using a hemocytometer. (**E**) Immunoblot confirms regulated expression of the Ty1-tagged RBP42 protein in RBP42^Ty1^ cells. α-tubulin is loading control. (**F**) Schematic of experimental design. Expression of RBP42 was turned off on day 0 by removing tetracycline from the growth medium. Knock down of RBP42 was indicated by gray ramp.

To investigate the functional significance of RBP42 binding on target gene expression, we generated an RBP42 conditional knockdown slender bloodstream form cell line (RBP42^Ty1^) in which cell growth depends upon a tetracycline-regulated exogenously expressed Ty1-tagged version of RBP42 (Fig. 4A and D). Two antibiotic resistance genes replaced two native RBP42 alleles, which were confirmed by genomic PCR (Fig. 4B). Immunoblot analysis confirmed that only the tagged RBP42 protein is expressed in RBP42^Ty1^ cells (Fig. 4C). In the presence of tetracycline, added to the growth media, RBP42^Ty1^ cells grew normally. However, loss of RBP42, in the absence of tetracycline, caused RBP42^Ty1^ cells to stop dividing after 2 days, as reflected by the growth curve, and eventual death (Fig. 4D). Immunoblot and immunofluorescence microscopic analyses confirmed the expected reduction of tagged-RBP42 protein in RBP42^Ty1^ cells (Fig. 4E and see Fig. S5a at https://doi.org/10.6084/m9.figshare.21737321). RBP42 knockdown triggered marked phenotypic alterations, with cells exhibiting abnormal shape, and containing multiple nuclei (see Fig. S5a at https://doi.org/10.6084/m9.figshare.21737321). Cell cycle analysis by flow cytometry reveals large increase in multinucleated (>2) cells; ~30% of cells following 2 days of RBP42 knockdown, compared to only ~2% of normally growing cells, indicating apparent cytokinesis defects (see Fig. S5b at https://doi.org/10.6084/m9.figshare.21737321). RBP42 knockdown triggered similar phenotypic alterations of procyclic forms (29), indicating its essential role in both mammalian and insect stages of the parasite. To seek answers to how RBP42 regulates *T. brucei* gene expression, we measured global changes in cellular transcriptome, proteome, and metabolome in RBP42^Ty1^ cells following loss of RBP42 (Fig. 4F).

### Loss of RBP42 has minimal effect on target mRNA abundance

To determine the effect of RBP42 on its target mRNA stability, we measured transcriptome levels (mRNA-seq) in RBP42^Ty1^ cells before and after 1, 2, and 3 days of RBP42 depletion (Fig. 4F). cDNA libraries, representing poly(A)^+^ RNA from triplicate samples of all 4 days were deep sequenced to obtain quantitative mRNA measurements (see Fig. S6a at https://doi.org/10.6084/m9.figshare.21737321). Principal component analysis of mRNA-seq data shows robust clustering of samples from same day, and clear separations of clusters from all 4 days, confirming reproducibility of our measurement (see Fig. S6b at https://doi.org/10.6084/m9.figshare.21737321). As expected, there is >10-fold drop in RBP42 mRNA levels starting from day 1 (see Fig. S6c at https://doi.org/10.6084/m9.figshare.21737321). Differential expression analysis, using DESeq2 (41), shows 208 mRNAs were significantly changed (adjusted *P* value < 0.01) following loss of RBP42: 111 upregulated (32 mRNAs > 2-fold) and 97 downregulated (only 2 mRNAs < 2-fold) (see Table S1 at https://doi.org/10.6084/m9.figshare.21737321). Upregulated mRNAs encode many cell membrane-associated proteins, including Variant Surface Glycoproteins and membrane transport proteins; downregulated mRNAs encode a number of proteins involved in metabolic processes.

However, loss of RBP42 did not elicit any clear, significant effect on abundances of its “most reliable” mRNA target set (see Fig. S7a and b at https://doi.org/10.6084/m9.figshare.21737321). Out of 183 mRNA targets, 133 were slightly upregulated (to a maximum of 1.3-fold, 12 of them with adjusted *P* < 0.01), and 49 were marginally downregulated (to a minimum of 0.8-fold, 11 of them with adjusted *P* < 0.01) (see Fig. S7c at https://doi.org/10.6084/m9.figshare.21737321). Therefore, we conclude that RBP42 is unlikely to control the cytoplasmic turnover of its target mRNAs.

### Loss of RBP42 leads to decreased levels of target mRNA-encoded protein

To assess the effect of RBP42 on mRNA translation, we measured overall protein synthesis rate before (day 0) and after 1 (day 1) and 2 (day 2) days of RBP42 depletion, using a recently developed non-radioactive method known as the surface sensing of translation (SUnSET) (42), which relies on the principle that puromycin, owing to its structural analogy to tyrosyl t-RNA, is incorporated into elongating peptide chains. By detecting newly synthesized puromycin incorporated peptides, with a monoclonal anti-puromycin antibody in immunoblot assays, we evaluated cellular translational activity. Total lysates from puromycin-treated cells detected a trail of puromycin-incorporated newly synthesized peptides (see Fig. S8a at https://doi.org/10.6084/m9.figshare.21737321). No signal was detected in control lysates, prepared from untreated cells, and therefore ruling out the possibility of non-specific antibody binding to existing peptides. We observed ~25% diminished rate of protein synthesis following 2 days of RBP42 knockdown (see Fig. S8b at https://doi.org/10.6084/m9.figshare.21737321). Reduced protein synthesis was also observed in procyclic forms following loss of RBP42 (29), indicating its possible role on mRNA translation in both mammalian and insect stages of the parasite.

To determine the specific effect of RBP42 on its target mRNA translation, we measured proteome levels in RBP42^Ty1^ cells before (day 0) and after 2 days of RBP42 depletion (day 2) using Isobaric Tags for Relative and Absolute Quantitation (iTRAQ)-based LC/MS/MS method. Total cellular proteins from eight samples, four replicates of each condition, were labeled with eight unique iTRAQ reagents and analyzed (see Fig. S9a at https://doi.org/10.6084/m9.figshare.21737321). All eight samples exhibit similar iTRAQ intensity distribution, with a median value ~100, indicating robust, reproducible measurement (see Fig. S9b at https://doi.org/10.6084/m9.figshare.21737321). As expected, there is >5-fold drop in RBP42 protein following 2 days of depletion (see Fig. S9c and d at https://doi.org/10.6084/m9.figshare.21737321). Unbiased clustering of proteomes revealed that replicate samples of RBP42 depleted cells (day 2) cluster separately from replicate samples of normally growing RBP42 repleted cells (day 0), indicating reproducibility of iTRAQ quantitation (see Fig. S9e at https://doi.org/10.6084/m9.figshare.21737321). Because of moderate-to-high positive correlation among replicate samples, analysis was performed by combining replicate data sets.

**Fig 5:**
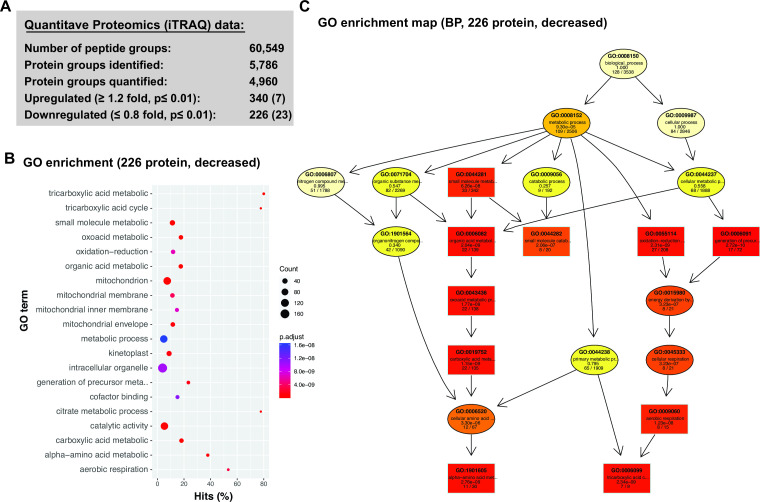
Loss of RBP42 reduces the levels of enzymatic proteins of central carbon and energy metabolic pathways. (**A**) iTRAQ quantitative proteomics of total cellular proteins from RBP42^Ty1^ cells before (day 0) and 2 days after (day 2) RBP42 knockdown. The number of protein groups with significantly (*P* ≤ 0.01, by paired sample *t*-test) changed levels (increased, ≥1.2-fold; decreased, ≤0.8-fold) are shown. The number of upregulated and downregulated proteins that are in iCLIP “most reliable” data set are shown in parenthesis. (**B**) Gene ontology (GO) enrichment analysis of 226 significantly downregulated proteins following loss of RBP42. Dot plot showing top 20 enriched GO term. The *x*-axis represents percent hits, which is the ratio of the number of proteins in the 226 decreased set to the number of all annotated proteins with same GO term. The sizes of the dots represent the number of downregulated proteins associated with the GO term. The colors of the dots represent adjusted *P* values (BH). (**C**) GO graph showing significantly enriched GO terms in the Biological Processes category of the 226 downregulated proteins (using TopGO). Each node marks a GO term, and each arrow indicates an “is-a” relationship. Boxes indicate 10 most significant nodes. Increasing coloring toward red represents increasing significance levels. The GO descriptions of each node along with significance levels and ratio of hits over total are also shown.

We observed major alterations in cellular proteome following loss of RBP42. Significant changes (*P* value < 0.01) were observed for 1,650 proteins (~30% of quantified proteome), of which 340 proteins showed ≥1.2-fold upregulation, and 226 proteins showed ≤0.8-fold downregulation (Fig. 5A and see Table S1 at https://doi.org/10.6084/m9.figshare.21737321). Of the 183 “most reliable” RBP42-target mRNA, identified by the iCLIP, our proteomic analysis quantified 178 mRNA-encoded proteins. Majority of the target-encoded proteins were downregulated; 109 proteins, of which 23 proteins <0.8-fold, in contrast to 69 upregulated proteins, of which 7 proteins >1.2-fold (see Fig. S10 at https://doi.org/10.6084/m9.figshare.21737321). The noticeable changes in RBP42-target mRNA-encoded proteins following loss of RBP42, but not target mRNA abundance, indicate a possible translational regulatory role of RBP42 in *T. brucei* gene expression. Although it is possible that RBP42 exerts distinct translational regulations, both positive and negative on discrete sets of mRNA targets, akin to human antigen R (HuR) protein (43, 44), for the majority of its targets, RBP42 acts as a positive regulator of translation. Since RBP42-targets include several RBPs with known post-transcriptional regulatory roles, the observed changes in global transcriptome (see Table S1 at https://doi.org/10.6084/m9.figshare.21737321) following loss of RBP42 is most likely a secondary effect.

To evaluate the effects of loss of RBP42 on specific cellular processes, we performed GO term enrichment analysis of both upregulated and downregulated proteins (Fig. 5A). Many of the 340 most significant upregulated proteins (>1.2-fold, *P* < 0.01) are involved in membrane lipid and GPI anchor synthetic process, as well as membrane transporters, and show enrichment of a few general (higher level) GO terms. These include carbohydrate derivative biosynthetic process (GO:1901137, *P* 4.41e−7), and lipid metabolic process (GO:0006629, *P* 3.27e−6). In contrast, the 226 most significant downregulated proteins (<0.8-fold, *P* < 0.01) show many enriched GO terms, both general (higher level) and specific (lower level), with high significance (*P* < 1e−05) that are associated with primary metabolism (Fig. 5B). Some very specific categories include tricarboxylic acid cycle (GO:0006099, *P* 2.34e−09), alpha amino acid metabolic process (GO:1901605, *P* 2.76e−09), and aerobic respiration (GO:0009060, *P* 1.23e−08). To obtain a meaningful biological interpretation, we analyzed these enriched GO terms using TopGO algorithm (45), which uses underlying GO graph topology to improve GO group scoring and reduce redundancy. The resulting GO graph of the Biological Process (BP) category, illustrated in Fig. 5C, shows clear downregulation of the central carbon and energy metabolic pathway following loss of RBP42.

**Fig 6:**
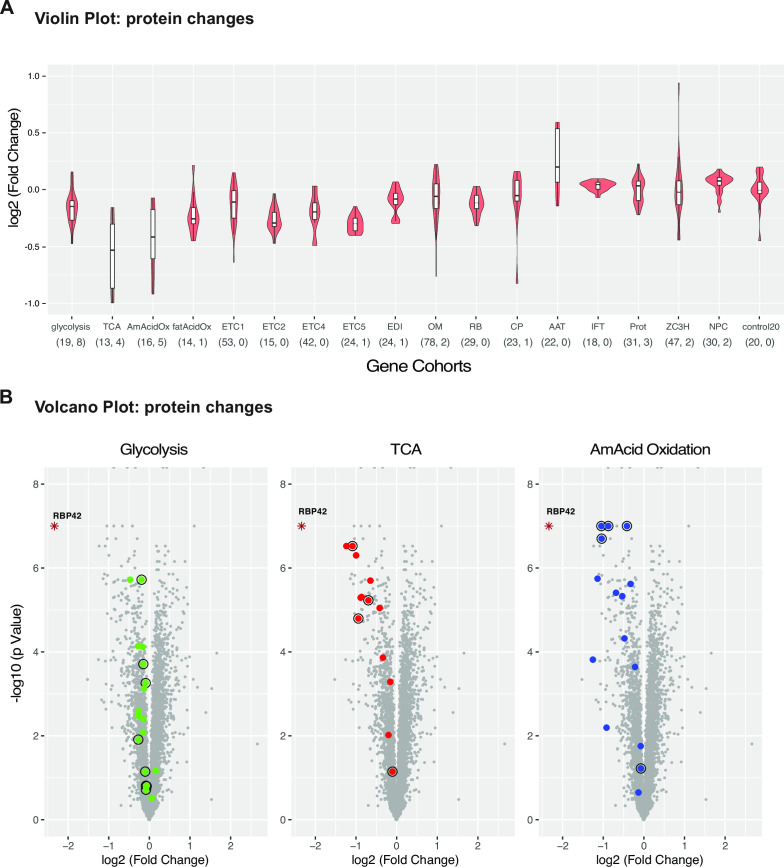
Loss of RBP42 causes significant downregulation of enzymatic proteins involved in glycolysis, TCA cycle, and amino acid oxidation. (**A**) Violin plots showing protein expression changes (iTRAQ intensity, log_2_) of gene cohorts following RBP42 knockdown. White box represents 25th to 75th percentile with the horizontal line as the median, and the whiskers extend 1.5 times the interquartile range. AAT, amino acid transporters; control20, a cohort of 20 randomly sampled proteins; CP; carrier proteins; EDI, editing complex; ETC1-5, mitochondrial respiratome complexes; IFT, intraflagellar transport; NPC, nuclear pore complex; OM, mitochondrial outer membrane proteins; Prot, proteasome; RB, RNA-binding complex; ZC3H, zinc finger family proteins. The number of proteins in each cohort and the corresponding numbers in iCLIP “most reliable” data set are shown in parenthesis. (**B**) Volcano plots showing changes in protein levels (log_2_ fold changes) versus significance *P* values (−log10) for all (~5,000) quantified proteins (iTRAQ) after 2 days of RBP42 knockdown. Individual proteins that constitute glycolysis, TCA, and alpha amino acid oxidation (AmAcid Oxidation) cohorts are color-coded; all other proteins are shown in gray. Significance *P* values are estimated by paired sample *t*-tests. Candidate proteins that are in iCLIP “most reliable” data set are outlined.

To examine which specific processes are mostly affected, we compiled, using published data set (23, 25, 46), a set of gene cohorts associated with central carbon and energy metabolic pathway. Analysis shows downregulation of many metabolic cohorts, but not control cohorts (Fig. 6A). The two most affected cohorts are tricarboxylic acid cycle (TCA) and alpha amino acid oxidation. We observed significant downregulation of enzymatic proteins that are part of the glycolytic, TCA cycle, and the alpha amino acid oxidation processes (Fig. 6B). As anticipated, the levels of mRNAs encoding these enzymes did not show any significant changes (see Fig. S11 at https://doi.org/10.6084/m9.figshare.21737321). Although the existing metabolic model for slender bloodstream form does not support mitochondrial substrate-level or oxidative phosphorylation-mediated ATP production, recent proteomic studies revealed that most, if not all, enzymes involved in the production of succinate and acetate are expressed (47 – 49). Importantly, several of these enzymes are also essential (47, 50, 51). For example, we observed noticeable downregulation of these essential enzymes following loss of RBP42: PDH-E2 (Tb927.10.7570), 0.54-fold; TDH (Tb927.6.2790), 0.69-fold; α-KDE2 (Tb927.11.11680), 0.52-fold; and SCoAS β subunit (Tb927.10.7410), 0.55-fold. Identification of glucose-derived metabolic intermediates produced in the “succinate and acetate branches” of the TCA cycle in slender bloodstream form mitochondria further supports the activity of these enzymes (48). Downregulation of these essential pathways, therefore, is expected to perturb cellular central carbon metabolism.

### Loss of RBP42 impairs cellular central carbon and energy metabolism

**Fig 7:**
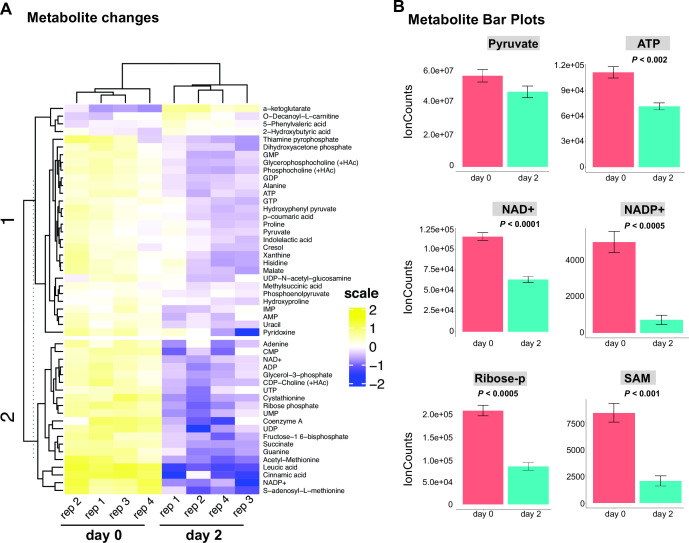
Loss of RBP42 alters cellular metabolic profile. (**A**) Heatmap view of metabolite levels following RBP42 knockdown. Metabolomes were quantified using LC-MS and metabolites were identified with known standards. Metabolites with significantly changed levels (*P* ≤ 0.05) are shown. Four replicate samples prepared from RBP42^Ty1^ cells before (day 0) and after 2 days of RBP42 knockdown (day 2) are indicated at the bottom. Metabolites are labeled on the right. The abundance of each metabolite is log_2_ transformed and mean-centered with blue being less abundant and yellow more abundant. The dendrograms are based on hierarchical clustering. (**B**) In the absence of RBP42 protein, several important molecules that determine cellular energy and redox states are significantly reduced. Bar plots showing mean ± standard error of signal intensities of six compounds before (red, day 0) and after (green, day 2) RBP42 knockdown. Significance *P* values, estimated by paired sample *t*-test, are shown.

To determine the effect of RBP42 on cellular metabolism, we measured levels of intracellular metabolites, including intermediates from glycolysis and TCA cycle, organic acids, and nucleotides. We reasoned that downregulation of the many metabolic enzymes following loss of RBP42 will alter the levels of these metabolic intermediates. Using mass spectrometry method, we generated metabolite profile of RBP42^Ty1^ cells before and after 2 days of RBP42 depletion. Compounds were extracted from four replicate samples of each condition and analyzed to measure quantitative changes in intermediary metabolites of glycolysis, TCA cycle, amino acids, and nucleotides. Analysis revealed noticeable reduction in many glucose-derived metabolites; out of a total of 138 quantified metabolites, 40 show significant decrease (*P* < 0.05) (Fig. 7A and see Table S1 at https://doi.org/10.6084/m9.figshare.21737321).

We observed noticeable (~20%) decline in pyruvate (Fig. 7B), which is the major end product of catabolized glucose in slender bloodstream form. Pyruvate also serves as a major source of alanine, which declined 35% following loss of RBP42. We also observed large decreases in oxaloacetate, malate, and succinate that are produced from the “succinate branch” of the TCA cycle. Oxaloacetate-derived aspartate is a known precursor of pyrimidine synthesis via dihydroorotate (52). Significant decreases are also observed in purine and pyrimidine nucleotides. In the absence of *de novo* synthesis, trypanosomes rely on purine salvage (53), which requires ribose 5-phosphate that is synthesized via the oxidative branch of pentose phosphate pathway (54). We observed ~60% decrease in ribose 5-phosphate, indicating that dysregulation of glucose metabolism also caused aberrations in pentose phosphate pathway. In addition to a steep decrease in ATP (~40%), we observed significant decreases in critical cofactors, NAD and NADP, and the methyl donor S-adenosyl methionine (Fig. 7B), all of which are essential for normal cellular metabolic activities. Taken together, these results show that RBP42 ensures proper regulation of core metabolic enzymes, which is critical for the parasite’s survival in the diverse nutritional environments encountered throughout its life cycle.

## DISCUSSION

**Fig 8:**
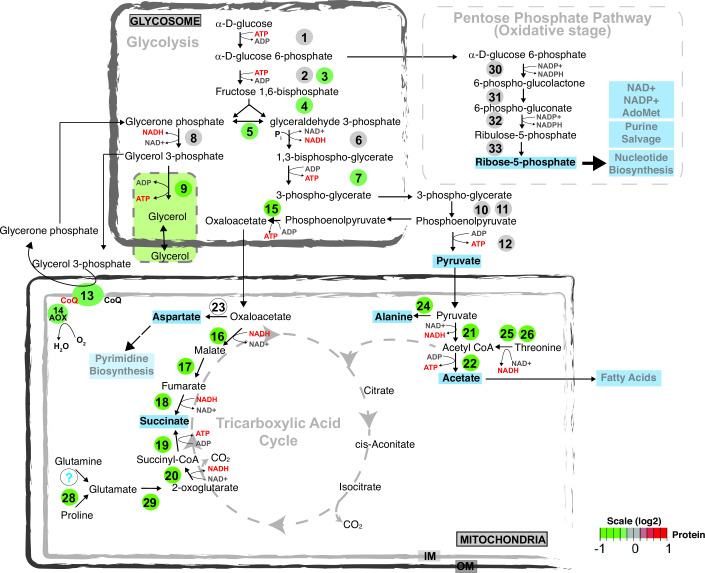
Changes in energy metabolic pathway following loss of RBP42. Schematic showing current consensus energy metabolic pathways that are active in slender bloodstream form trypanosomes. Dashed arrows indicate enzymatic steps for which no evidence of metabolic flux is available. Major intermediate metabolites that are either excreted out of the cell or utilized for biosynthesis of fatty acids or nucleotides are shown in bold. Numbered, colored circles indicate enzymes that are quantified in this study. Changes (log_2_ scale) in protein amounts are denoted using green-to-red color ramp. Enzymes are 1, hexokinase (HK); 2, glucose 6-phosphate isomerase (PGI); 3, phosphofructo kinase (PFK); 4, fructose bisphosphate aldolase (ALD); 5, triose-phosphate isomerase (TIM); 6, glyceraldehyde-3-phosphate dehydrogenase (GAPDH); 7, phosphoglycerate kinase (PGK); 8, NADPH-dependent glycerol-3-phosphate dehydrogenase (G3PDH, glycosomal); 9, glycerol kinase (GK); 10, phosphoglycerate mutase (PGAM); 11, enolase (ENO); 12, pyruvate kinase (PYK1); 13, FAD-dependent glycerol-3-phosphate dehydrogenase (G3PDH, mitochondrial); 14, alternative oxidase (AOX); 15, phosphoenolpyruvate carboxykinase (PEPCK); 16, malate dehydrogenase, mitochondrial (mMDH); 17, fumarate hydratase, mitochondrial (FHm); 18, NADPH-dependent fumarate reductase, mitochondrial (FRDm1); 19, succinyl-CoA synthetase (SCoAS); 20, 2-oxoglutarate dehydrogenase (2-OGDH); 21, pyruvate dehydrogenase (PDH); 22, acetate:succinate CoA-transferase (ASCT); 23, aspartate aminotransferase, mitochondrial (mASAT); 24, alanine aminotransferase (ALAT); 25, threonine dehydrogenase (TDH); 26, 2-amino-3-ketobutyrate CoA ligase (AKCT); 28, proline dehydrogenase, mitochondrial (PRODH); 29, glutamate dehydrogenase (GDH); 30, glucose-6-phosphate 1-dehydrogenase (G6PD); 31, 6-phosphogluconolactonase (6PGL); 32, 6-phosphogluconate dehydrogenase (6PGDH); 33, ribose-5-phosphate isomerase (RPI).

RBP-mediated post-transcriptional regulation play key roles in trypanosome gene expression. Although several important RBPs have been studied to date, details of their regulatory roles remain elusive. Here, employing a detailed analysis that combines *in vivo* RNA target identification with global transcriptomic, proteomic, and metabolic profiling, we provide evidence that RBP42 acts as a critical regulator of *T. brucei* central carbon and energy metabolism in mammalian-infective slender bloodstream forms (Fig. 8 and see Fig. S12 at https://doi.org/10.6084/m9.figshare.21737321). Our analysis reveals that RBP42 targets mRNAs encoding enzymes involved in core metabolic processes. This finding is consistent with previously identified targets in the fly-infective procyclic forms (29), indicating that RBP42 plays a conserved role in regulating metabolic genes in both stages of the parasite.

Using a very stringent iCLIP criteria, we identified 189 transcripts as “most reliable” RBP42 targets. Although iCLIP is extremely powerful in identifying crosslink sites at single nucleotide resolutions, various technical limitations hinder an accurate cataloging and precise enrichment estimation of all mRNA targets relative to RNA abundance (36). This is particularly challenging for genes that are present in multiple identical or almost identical copies in the genome (55). For example, alpha tubulin mRNAs, a very abundant mRNA, is not identified as RBP42 target in our “most reliable” set, contrary to a recent *in vivo* mRNP capture study that indicated that RBP42 is associated with alpha tubulin mRNAs (56). Only future studies with improved techniques and computational strategies can reveal the true comprehensive set of RBP42 interacting transcripts.

Our study revealed that RBP42 binds within the coding region of target mRNAs. Analysis of RBP42 crosslink sites on mRNA shows preference to a hexanucleotide sequence motif centered around a di-cytidine (Fig. 2D). However, a majority of the crosslink sites on RNA are devoid of this motif, raising the question of how RBP42 associates with specific sets of mRNAs. Since *in vitro* assays show RBP42 binding to mutated target RNA (Fig. 2E), albeit with reduced affinity, it is possible that RBP42 interacts with cryptic RNA motifs, distributed on target mRNA after initial loading, or recruited to some target mRNA via protein-protein interactions.

Having defined RBP42’s RNA target set, we were motivated by curiosity to understand its impact on the regulation of these genes. We focused on the transcript and protein levels of the significant subset of iCLIP-identified targets following loss of RBP42, using a conditional knockdown strategy. Depletion of RBP42 had little effect on steady-state levels of target mRNAs, but major effect on the target proteome, indicating a possible translational regulatory role of RBP42. This is consistent with polysomal association of RBP42 observed in a previous study (29). Since no major changes are observed in the steady-state levels of RBP42-target transcripts following loss of RBP42, we speculate that additional factors are involved that possibly sequester these target transcripts in mRNA storage granules.

Loss of RBP42 caused a moderate reduction in overall cellular translation rate, ~15% on day 1, and ~25% on day 2 (see Fig. S8 at https://doi.org/10.6084/m9.figshare.21737321). This cell-wide reduction in translation rate can be a result of either a uniform partially reduced translation rate of each mRNA, ~25% on day 2 in this instance, or severely reduced translation rate of a subset of mRNAs while the other mRNAs are translated normally. Our data suggest that there are more pronounced translational defects for the RBP42-target mRNAs. However, RBP42-targets include several translation factors of which three are known to be essential from a genome-scale “loss of fitness” study (57) (EIF3L, Tb973.10.4640; EIF3B, Tb927.5.2570; EIF4A1; Tb927.9.4680). Even a slight reductions in these essential translation factors are expected to impair translations of the majority of cellular mRNAs. Therefore, ~25% reduction in overall translation rate after 2 days of RBP42 knockdown is probably a combined effect, a more pronounced direct effect of loss of RBP42 on its target mRNAs, plus a secondary effect on other non-target mRNAs because of diminishing essential translation factors. Dissecting this is an important future goal.

Our analysis indicates that loss of RBP42 caused clear downregulation of many enzymes involved in core metabolic processes. Along with glycolysis, the two most affected pathways are mitochondria-resident interconnected energy metabolic pathways, i.e., TCA cycle and amino acid oxidation. It is increasingly recognized that many cellular mRNAs are translated within subcellular domains that allow precise localization and regulation of the newly made proteins (58). RBP42 may permit translocation of mitochondrial-resident protein synthesis, while protecting mRNA from degradation. Similar coordinated control of mitochondrial respiratome components is proposed for *T. brucei* ZC3H39/40 RNA-binding complex (25). RBP42 is known to be phosphorylated (59), which is a well-recognized mechanism that controls the activity of many RBPs (60 – 62). It is possible that phosphorylation of RBP42 is regulated by nutrient availability, which in turn may regulate its activity as a translational regulator to fine-tune cellular metabolic activity.

Importantly, loss of RBP42 resulted in significant reduction of many intermediary metabolites. Energy metabolism, i.e., production and utilization of ATP, is a highly complex, dynamic process involving numerous factors that respond to intra- and extra-cellular signals, nutrient availability, and cellular physiological and developmental status. Slender bloodstream form *T. brucei* relies mainly on glucose as the major carbon source and current metabolic model suggests that ATP is exclusively produced via glycolysis, pyruvate being the major (~85%) end product (48). The high ATP demand of proliferating cells is achieved by ~10-fold greater glycolytic rate in slender bloodstream form, compared to procyclic form. However, recent proteomic studies have confirmed that although mitochondrial oxidative- and substrate-level phosphorylation is not utilized for ATP production, various mitochondrial activities are essential for slender form survival. Metabolomic studies confirmed that slender form excretes significant levels of alanine, acetate, and succinate that are produced in the mitochondria. Mitochondrial production of acetate is essential for the slender bloodstream form *T. brucei* (47). Glucose-derived pyruvate and threonine are two main sources of acetate, which is produced via the “acetate branch” of TCA cycle. Therefore, marked reduction of both pyruvate dehydrogenase (PDH) and threonine dehydrogenase (TDH), following RBP42 knockdown, is expected to severely weaken acetate production.

RBP42 knockdown reduced the levels of many glycolytic enzymes. This modulation of glycolysis may impede the proper function of the oxidative branch of pentose phosphate pathway and therefore reduce ribose 5-phosphate and nucleotide salvage pathways. A marked, ~40%, drop in ATP level was observed. Cellular metabolic activities primarily rely on ATP as energy carrier that drives anabolic reactions critical for cell survival and proliferation. Importantly, ATP also works as structural precursor of important cellular cofactors, including NAD, NADP, and the methyl donor S-adenosyl methionine (SAM). Therefore, a decrease in ATP level is expected to cause widespread disruption of metabolic activity. For example, a decline in SAM is expected to cause major alterations in activities of many methylation-dependent RBP. Absence of protein arginine methylation is known to cause striking changes in cellular energy metabolism (27).

In conclusion, it is clear that RBP42 allows proper expression of metabolic enzymes involved in *T. brucei* central carbon and energy metabolism. By undertaking a broad approach, we show that RBP42-mediated regulation of metabolic networks is critical for the parasite. Importantly, RBP42 homologs are also present in other trypanosomatid organism, including parasitic *T. cruzi* and *Leishmania spp* (63). We anticipate that RBP42 homologs play similar metabolic regulatory roles in these related organisms. Although RBP42’s precise mode of action remains to be discovered, our analysis hints an apparent translational regulatory role of RBP42.

## MATERIALS AND METHODS

### *T. brucei* strain and growth analysis

*T. brucei* Lister 427 bloodstream form wild-type cells, single marker (SM) cells (64), and all stable transgenic cell lines were grown in HMI-9 medium supplemented with 10% fetal bovine serum and 10% serum plus at 37°C in a humidified incubator containing 5% CO_2_. SM cells that co-express T7 RNA polymerase and the Tet repressor with *NEO* resistance gene were maintained with 2.5 µg/mL G418. Recombinant DNA constructs were introduced by nucleofection using Amaxa Human T-solution following the manufacturer’s instruction. Transgenic cell lines were selected and grown by the addition of puromycin, phleomycin, and blasticidin as required at 0.1, 1.25, and 5 µg/mL, respectively. Homozygous triple-Ty1-tagged RBP42 cell line was generated by N-terminal epitope tagging of both RBP42 alleles at the native loci. RBP42 conditional knockdown cell line (RBP42^Ty1^) was generated by replacing two native RBP42 alleles with *BLE* and *PAC* selectable marker cassettes and introducing an ectopic inducible Ty1-tagged RBP42 allele. The inducible expression construct was introduced into a single RBP42 allele null strain prior to deletion of the second RBP42 allele, while maintaining RBP42 expression by addition of tetracycline to the culture media. RBP42^Ty1^ cells were grown in the presence of 1 µg/mL tetracycline to maintain inducible expression of exogenous RBP42 transgene. To shut down the expression of the exogenous RBP42 transgene, cells were washed twice to remove tetracycline and resuspended in culture medium lacking tetracycline. Cultures were seeded at 1 × 10^5^ cells/mL and growth analysis was carried out by counting cell density on a hemocytometer.

### Plasmid constructs

Epitope tagging of RBP42 alleles at the native loci was carried out using PCR-based strategy (65). Two PCR-generated DNA modules, one with *NEO* resistance gene and the other with *PAC* resistance gene, were used in two successive rounds of transfection and selection to tag two native RBP42 alleles. The ectopic inducible construct was made by inserting Ty1-tagged RBP42 open reading frame into plasmid pAD74 (66). The resulting construct was linearized using NotI restriction enzyme to facilitate homologous recombination into rRNA loci. A *BLA* resistance gene within the construct allowed selection of stable cell lines. To replace endogenous RBP42 alleles, knockout gene cassettes were generated by cloning 500 bp upstream and 1,000 bp downstream sequences to flank *BLE* and *PAC* resistance genes. Knockout cassettes were released by restriction enzyme digestion prior to transfection.

### Crosslink immunoprecipitation (CLIP)

CLIP was carried out with minor modifications of previously published method (29). Briefly, slender bloodstream forms (0.5–0.8 × 10^6^ cells/mL) were harvested and washed once with cold trypanosome buffer saline (TBS: 137 mM NaCl, 2.7 mM KCl, 10 mM Na_2_HPO_4_, 1.8 mM KH_2_PO_4_, 20 mM glucose, pH 7.4). Cells, resuspended in 6 mL TBS to a concentration of ~5 × 10^7^ cells/mL, were transferred to a 100-mm Petri dish, placed on an ice tray, and UV-irradiated (254 nm) once with 150 mJ/cm^2^ or 300 mJ/cm^2^ dose in Stratalinker 1800 (Stratagene, La Jolla, CA) UV light source. Cells were rapidly pelleted, snap-frozen in liquid N_2_, and stored at −80°C in small aliquots. Crosslinked RBP42-RNA complexes were immunopurified from cellular extracts using antibodies attached to magnetic beads (Dynabeads): anti-RBP42 antibody to Protein G beads and anti-Ty1 (BB2) antibody to sheep anti-mouse IgG beads. Captured protein-RNA complexes were washed extensively and analyzed by gel chromatography.

### iCLIP-Seq

iCLIP-Seq libraries were prepared following the published method (67). Prior to immunoprecipitation, cellular extracts were treated with titrated amount of RNase I to generate median of 40–80 nt long RNAs. After high-salt stringent washing, RNAs on beads were ligated to an adapter at the 3′ end, and radioactively labeled on the 5′ end. Protein-RNA complexes, run on 4–12% NuPAGE Bis-Tris gel (Invitrogen, Waltham, MA) and transferred on to Nitrocellulose membrane, were detected using X-ray film. Complexes in the range of 20–40 kDa above RBP42 were excised, and RNA was recovered by proteinase K digestion. Reverse transcription of RNA was performed using primers with two cleavable adapter regions separated by a *Bam*HI site, as well as a barcode to mark unique cDNA molecules. cDNAs were size-selected into two sizes, 80–100 nt as low (L) and 100–150 nt as high (H), using denaturing gel electrophoresis, circularized by CircLigase II ssDNA ligase (Lucigen, Middleton, WI). Subsequently, cDNAs were linearized by BamHI digestion, PCR-amplified, and sequenced on the Illumina NextSeq platform.

iCLIP-Seq data were analyzed using published “analysis pipeline” (68). Raw sequence reads, quality checked using FastQC (www.bioinformatics.babraham.ac.uk/projects/fastqc), were trimmed to remove adapter and barcode sequences. The barcode sequence was assigned to each read, using Flexbar, as unique molecular identifier (UMI) that was later used to eliminate PCR duplicates (69, 70). Sequence reads of at least 15 nt in length were mapped to a hybrid genome consisting of *T. brucei* 927 genome assembly (version 45) plus *T. brucei* Lister427 telomeric contigs using STAR aligner with parameters set to search only unique alignment, with less than 4% mismatched bases (71). Following removal of PCR duplicates using UMI-tools, each unique cDNA molecule was counted as an independent crosslink event. Crosslinked sites, the nucleotide preceding the cDNA start, were extracted using BEDTools suit (72). Significant crosslink sites were determined using PureCLIP cluster finding algorithm (38). Crosslink sequences were extracted by adding 5 nt flanking sequences to crosslink sites to obtain 11 nt crosslinking regions. Crosslink sequence motif discovery was performed using the MEME suit (https://meme-suite.org/tools/meme), with the parameters—classic mode, zero or one occurrence per sequence, search given strand only, minimum width 4, maximum width 10.

### qRT-PCR analysis

CLIP-purified RNA, as described above, was subjected to reverse transcription (RT) with random hexamer primers and iScript reverse transcriptase (Bio-Rad, Hercules, CA). Quantitative reverse transcription PCR (qRT-PCR) was performed in a CFX96 Touch Real-Time PCR detection system using primer pairs targeted at specific transcripts (see Table S1 at https://doi.org/10.6084/m9.figshare.21737321). Data were analyzed using the CFX Manager 3.0 software. Bar plots were generated using mean ± standard errors.

### mRNA-Seq

mRNA-Seq libraries were prepared from poly(A)^+^-containing RNA, captured by two rounds of oligo-d(T)_n_-bead selection of 10 µg total RNA, following Illumina small RNA library preparation method. Libraries were sequenced on the Illumina NextSeq platform. Sequence reads were mapped to the *T. brucei* 927 genome assembly version 45 using Bowtie2 (v2.3.5). The mapped reads were then converted to gene expression values and analyzed using DESeq2 (41) and Cuffdiff2 (73) (see Table S1 at https://doi.org/10.6084/m9.figshare.21737321). Volcano and violin plots were generated using expression value (log_2_ fold change) and *P* value in R.

### SUnSET assay

Cellular protein synthesis rate was analyzed using SUnSET method (42). RBP42^Ty1^ cells (2 × 10^7^ cells/assay), from before (day 0) and after 1 and 2 days (day 2) of RBP42 knockdown, were incubated with puromycin (10 µg/mL) for 30 min at 37°C. RBP42^Ty1^ cells without puromycin act as control. Parasites were harvested and washed with ice-cold TBS, and total proteins were extracted using RIPA buffer (20 mM Tris-HCl at pH 7.5, 150 mM NaCl, 1 mM EDTA, 1 mM EGTA, 1% NP-40, 1% Na-deoxycholate, and protease inhibitor cocktail). Puromycin incorporation was determined by immunoblot analysis using monoclonal antibody against puromycin (Kerafast, Boston, MA) and quantified using ImageJ.

### iTRAQ quantitative proteomics

Quantitative proteomics using iTRAQ method (74) were performed at the Center for Advanced Proteomics Research (NJMS, Rutgers Biomedical and Health Sciences, Newark, NJ). RBP42^Ty1^ cells (5 × 10^7^ cells/assay) were harvested before (day 0) and after 2 days (day 2) of RBP42 knockdown and washed with ice-cold TBS. Total proteins were extracted using lysis buffer containing 100 mM TEAB, 8M urea, and protease inhibitor cocktail. About 100 µg proteins, from four replicate samples of each condition, was reduced, alkylated, and trypsin digested before subjected to labeling with 8-plex iTRAQ reagents (AB Sciex, Framingham, MA). Peptides from day 0 replicates were labeled with iTRAQ tag—113, 114, 115, and 116—whereas peptides from day 2 replicates were labeled with iTRAQ tag—117, 118, 119, and 121. Subsequently, all labeled peptides from eight samples were pooled and fractionated using high pH RPLC liquid chromatography on ACQUITY UPLC system (Waters Corporation, Millford, MA). A total of 48 fractions were collected in 60 min gradient of Solvent A (20 mM HCOONH_4_, pH 10.0) and Solvent B (20 mM HCOONH_4_ in 85% ACN, pH 10.0) and pooled into 12 fractions that were subjected to LC-MS/MS analysis on an UltiMate 3000 RSLCnano coupled with Orbitrap Fusion Lumos Mass Spectrometer (Thermo Scientific). Peptides, ~1 µg from each fraction, were separated on a nano C18 column (Acclaim PepMap, 75 µm × 50 cm, 2 µm, 100 Å) using a 2-h non-linear binary gradient of mobile phase A (2% ACN and 0.1% formic acid) and mobile phase B (85% ACN and 0.1% formic acid) at a flow rate of 300 nL/min. Eluted peptides were introduced into Orbitrap Fusion Lumos system through a nanospray Flex ion source (Thermo Scientific, Waltham, MA) with the spray voltage of 2 kV and a capillary temperature of 275°C. The MS spectra was acquired in a positive mode. For MS1, peptide scan range was set to 375–1,500 with the resolution of 120,000. Peptides with charge-state of 2–7, and intensity greater than 5 × 10^3^ were selected for MS/MS scan in ion-trap using collision-induced dissociation (CID) with the collision energy of 35%. The dynamic exclusion is 60 s and the isolation window is 0.7 *m/z*. For SPS-MS3 scan, the precursor selection range was 400–1,200 with iTRAQ ion excluded. Ten SPS precursors were selected for MS3 scan in orbitrap with resolution of 50,000. High energy collision dissociation (HCD), with the collision energy of 65%, was used for iTRAQ tag quantitation.

The iTRAQ MS data were searched against UniProt *T. brucei brucei* (strain 927/4 GUTat10.1) database (8,579 proteins) using Sequest search engine on Proteome Discoverer (V2.4) platform. MS1 mass tolerance was set to 10 ppm and MS2 mass tolerance was 0.6 Da. iTRAQ 8-plex (K), iTRAQ 8-plex (N-terminal), and methylthio (C) were set as fixed modification, whereas oxidation (M) and iTRAQ 8-plex (Y) as variable modifications. Two missed cleavages are allowed in trypsin digestion. The reporter ion-based quantification workflow was chosen for data analysis; the CID spectra in MS2 were used for peptide identification and the HCD spectra in MS3 were used for iTRAQ quantitation. The false discovery rate for protein and peptide was set to 1% filtered with Percolator. The protein relative quantitation was calculated based on the ratio of (average in day 2 abundance)/(average in day 0 abundance) (see Table S1 at https://doi.org/10.6084/m9.figshare.21737321). Significance (*P* values) was computed using paired sample *t*-test.

### Metabolomics

Metabolomic profiling was performed at the Metabolomics Shared Resources Facility (Rutgers Cancer Institute of New Jersey, New Brunswick, NJ), following the published method (75). Briefly, cells (2 × 10^7^ cells/assay) were harvested as above. Metabolites were extracted with 1 mL of 40:40:20 mixture of methanol:acetonitrile:water plus 0.5% (vol/vol) formic acid on ice for 5 min. Following neutralization of formic acid by addition of 50 µL of 15% (m/vol) NH_4_HCO_3_, cleared extracts were collected by centrifugation at 15,000× *g* for 10 min and stored at −80°C. Metabolomic data, designed to capture intermediary metabolites in central carbon metabolism including glycolytic intermediates, TCA compounds, amino acids, nucleotides, and derivatives, were obtained using hydrophilic interaction liquid chromatography separation method coupled with mass spectrometry run in negative ionization mode. Each metabolite was identified by matching of accurate mass and retention time to synthetic standards. Metabolite bar plots were generated using mean ion counts ±standard errors. Significance (*P* value) was computed using paired sample *t*-test (see Table S1 at https://doi.org/10.6084 /m9.figshare.21737321).

### Flow cytometry

Cells (1 × 10^7^ cells/assay) were stained with Hoechst 33342 (1 μg/mL) in growth medium at 37°C for 10 min and run on a BD-LSR (Becton Dickinson, Franklin Lakes, NJ, USA). Data were analyzed using FlowJo.

### Fluorescence microscopy

Cells were fixed in 1% formaldehyde and adhered to slides coated with poly-L-lysine. RBP42 immunolocalization was performed using anti-RBP42 antibodies at 1:10,000 on cells permeabilized with 0.2% NP40 for 5 min at room temperature. FITC conjugated secondary antibodies are used at 1:1,000. Cells were mounted with Vectashield (Vector Laboratories, Newark, CA) containing DAPI and imaged using an Olympus BX61 microscope equipped with DAPI and FITC-sensitive filters and a Hamamatsu ORCA-ER camera.

### Gene ontology (GO) and pathway enrichment analysis

GO term enrichment analysis was performed using resources available from TriTrypDB.org database. REVIGO web tool was employed to summarize GO enrichment by eliminating redundant GO terms (76). Directed acyclic graph of enriched GO terms was produced using R Bioconductor topGO package (45). Pathway analysis was performed by R Bioconductor packages GAGE (77) and Pathview (78) using Kyoto Encyclopedia of Genes and Genomes database (79).

## Data Availability

All sequencing data (iCLIP-Seq, mRNA-Seq) are available from the NCBI BioProject database with accession number PRJNA734911. The iTRAQ proteomics data are available from the ProteomeXchange Consortium via the PRIDE partner repository with the data set identifier PXD026622. The metabolomics data are available at the NIH Common Fund’s National Metabolomics Data Repository (NMDR) website, the Metabolomics Workbench with project ID PR001151.

## References

[1] Keene JD . 2007. RNA regulons: coordination of post-transcriptional events. Nat Rev Genet 8:533–543. doi:10.1038/nrg2111 17572691

[2] Blackinton JG , Keene JD . 2014. Post-transcriptional RNA regulons affecting cell cycle and proliferation. Semin Cell Dev Biol 34:44–54. doi:10.1016/j.semcdb.2014.05.014 24882724 PMC4163074

[3] Bisogno LS , Keene JD . 2018. RNA regulons in cancer and inflammation. Curr Opin Genet Dev 48:97–103. doi:10.1016/j.gde.2017.11.004 29175729 PMC6489128

[4] Joshi A , Van de Peer Y , Michoel T . 2011. Structural and functional organization of RNA regulons in the post-transcriptional regulatory network of yeast. Nucleic Acids Res 39:9108–9117. doi:10.1093/nar/gkr661 21840901 PMC3241661

[5] Culjkovic-Kraljacic B , Borden KLB . 2018. The impact of post-transcriptional control: better living through RNA regulons. Front Genet 9:512. doi:10.3389/fgene.2018.00512 30455716 PMC6230556

[6] Büscher P , Cecchi G , Jamonneau V , Priotto G . 2017. Human African trypanosomiasis. Lancet 390:2397–2409. doi:10.1016/S0140-6736(17)31510-6 28673422

[7] Clayton CE . 2014. Networks of gene expression regulation in Trypanosoma brucei. Mol Biochem Parasitol 195:96–106. doi:10.1016/j.molbiopara.2014.06.005 24995711

[8] De Gaudenzi JG , Carmona SJ , Agüero F , Frasch AC . 2013. Genome-wide analysis of 3'-untranslated regions supports the existence of post-transcriptional regulons controlling gene expression in trypanosomes. PeerJ 1:e118. doi:10.7717/peerj.118 23904995 PMC3728762

[9] Vickerman K . 1985. Developmental cycles and biology of pathogenic trypanosomes. Br Med Bull 41:105–114. doi:10.1093/oxfordjournals.bmb.a072036 3928017

[10] Berriman M , Ghedin E , Hertz-Fowler C , Blandin G , Renauld H , Bartholomeu DC , Lennard NJ , Caler E , Hamlin NE , Haas B , Böhme U , Hannick L , Aslett MA , Shallom J , Marcello L , Hou L , Wickstead B , Alsmark UCM , Arrowsmith C , Atkin RJ , Barron AJ , Bringaud F , Brooks K , Carrington M , Cherevach I , Chillingworth T-J , Churcher C , Clark LN , Corton CH , Cronin A , Davies RM , Doggett J , Djikeng A , Feldblyum T , Field MC , Fraser A , Goodhead I , Hance Z , Harper D , Harris BR , Hauser H , Hostetler J , Ivens A , Jagels K , Johnson D , Johnson J , Jones K , Kerhornou AX , Koo H , Larke N , Landfear S , Larkin C , Leech V , Line A , Lord A , Macleod A , Mooney PJ , Moule S , Martin DMA , Morgan GW , Mungall K , Norbertczak H , Ormond D , Pai G , Peacock CS , Peterson J , Quail MA , Rabbinowitsch E , Rajandream M-A , Reitter C , Salzberg SL , Sanders M , Schobel S , Sharp S , Simmonds M , Simpson AJ , Tallon L , Turner CMR , Tait A , Tivey AR , Van Aken S , Walker D , Wanless D , Wang S , White B , White O , Whitehead S , Woodward J , Wortman J , Adams MD , Embley TM , Gull K , Ullu E , Barry JD , Fairlamb AH , Opperdoes F , Barrell BG , Donelson JE , Hall N , Fraser CM , Melville SE , El-Sayed NM . 2005. The genome of the African trypanosome Trypanosoma brucei. Science 309:416–422. doi:10.1126/science.1112642 16020726

[11] Siegel TN , Hekstra DR , Kemp LE , Figueiredo LM , Lowell JE , Fenyo D , Wang X , Dewell S , Cross GAM . 2009. Four histone variants mark the boundaries of polycistronic transcription units in Trypanosoma brucei. Genes Dev 23:1063–1076. doi:10.1101/gad.1790409 19369410 PMC2682952

[12] Queiroz R , Benz C , Fellenberg K , Hoheisel JD , Clayton C . 2009. Transcriptome analysis of differentiating trypanosomes reveals the existence of multiple post-transcriptional regulons. BMC Genomics 10:495. doi:10.1186/1471-2164-10-495 19857263 PMC2772864

[13] Clayton CE . 2002. Life without transcriptional control? From fly to man and back again. EMBO J 21:1881–1888. doi:10.1093/emboj/21.8.1881 11953307 PMC125970

[14] Kramer S , Carrington M . 2011. Trans-acting proteins regulating mRNA maturation, stability and translation in trypanosomatids. Trends Parasitol 27:23–30. doi:10.1016/j.pt.2010.06.011 20609625 PMC3070815

[15] Kolev NG , Ramey-Butler K , Cross GAM , Ullu E , Tschudi C . 2012. Developmental progression to infectivity in Trypanosoma brucei triggered by an RNA-binding protein. Science 338:1352–1353. doi:10.1126/science.1229641 23224556 PMC3664091

[16] Mugo E , Clayton C . 2017. Expression of the RNA-binding protein RBP10 promotes the bloodstream-form differentiation state in Trypanosoma brucei. PLoS Pathog 13:e1006560. doi:10.1371/journal.ppat.1006560 28800584 PMC5568443

[17] Bringaud F , Rivière L , Coustou V . 2006. Energy metabolism of trypanosomatids: adaptation to available carbon sources. Mol Biochem Parasitol 149:1–9. doi:10.1016/j.molbiopara.2006.03.017 16682088

[18] Smith TK , Bringaud F , Nolan DP , Figueiredo LM . 2017. Metabolic reprogramming during the Trypanosoma brucei life cycle. F1000Res 6:683. doi:10.12688/f1000research.10342.2 PMC5461901 28620452

[19] Acestor N , Zíková A , Dalley RA , Anupama A , Panigrahi AK , Stuart KD . 2011. Trypanosoma brucei mitochondrial respiratome: composition and organization in procyclic form. Mol Cell Proteomics 10:M110. doi:10.1074/mcp.M110.006908 PMC3186196 21610103

[20] Lamour N , Rivière L , Coustou V , Coombs GH , Barrett MP , Bringaud F . 2005. Proline metabolism in procyclic Trypanosoma brucei is down-regulated in the presence of glucose. J Biol Chem 280:11902–11910. doi:10.1074/jbc.M414274200 15665328

[21] Bakker BM , Michels PA , Opperdoes FR , Westerhoff HV . 1997. Glycolysis in bloodstream form Trypanosoma brucei can be understood in terms of the kinetics of the glycolytic enzymes. J Biol Chem 272:3207–3215. doi:10.1074/jbc.272.6.3207 9013556

[22] Haanstra JR , van Tuijl A , van Dam J , van Winden W , Tielens AGM , van Hellemond JJ , Bakker BM . 2012. Proliferating bloodstream-form Trypanosoma brucei use a negligible part of consumed glucose for anabolic processes. Int J Parasitol 42:667–673. doi:10.1016/j.ijpara.2012.04.009 22580731

[23] Trindade S , Rijo-Ferreira F , Carvalho T , Pinto-Neves D , Guegan F , Aresta-Branco F , Bento F , Young SA , Pinto A , Van Den Abbeele J , Ribeiro RM , Dias S , Smith TK , Figueiredo LM . 2016. Trypanosoma brucei parasites occupy and functionally adapt to the adipose tissue in mice. Cell Host Microbe 19:837–848. doi:10.1016/j.chom.2016.05.002 27237364 PMC4906371

[24] Capewell P , Cren-Travaillé C , Marchesi F , Johnston P , Clucas C , Benson RA , Gorman T-A , Calvo-Alvarez E , Crouzols A , Jouvion G , Jamonneau V , Weir W , Stevenson ML , O’Neill K , Cooper A , Swar N-R , Bucheton B , Ngoyi DM , Garside P , Rotureau B , MacLeod A . 2016. The skin is a significant but overlooked anatomical reservoir for vector-borne African trypanosomes. Elife 5:e17716. doi:10.7554/eLife.17716 27653219 PMC5065312

[25] Trenaman A , Glover L , Hutchinson S , Horn D . 2019. A post-transcriptional respiratome regulon in trypanosomes. Nucleic Acids Res 47:7063–7077. doi:10.1093/nar/gkz455 31127277 PMC6648352

[26] Bevkal S , Naguleswaran A , Rehmann R , Kaiser M , Heller M , Roditi I . 2021. An Alba-domain protein required for proteome remodelling during trypanosome differentiation and host transition. PLoS Pathog 17:e1009239. doi:10.1371/journal.ppat.1009239 33493187 PMC7861527

[27] Kafková L , Tu C , Pazzo KL , Smith KP , Debler EW , Paul KS , Qu J , Read LK . 2018. Trypanosoma brucei PRMT1 is a nucleic acid binding protein with a role in energy metabolism and the starvation stress response. mBio 9:e02430-18. doi:10.1128/mBio.02430-18 PMC6299225 30563898

[28] Liu B , Kamanyi Marucha K , Clayton C . 2020. The zinc finger proteins ZC3H20 and ZC3H21 stabilise mRNAs encoding membrane proteins and mitochondrial proteins in insect-form Trypanosoma brucei. Mol Microbiol 113:430–451. doi:10.1111/mmi.14429 31743541

[29] Das A , Morales R , Banday M , Garcia S , Hao L , Cross GAM , Estevez AM , Bellofatto V . 2012. The essential polysome-associated RNA-binding protein RBP42 targets mRNAs involved in Trypanosoma brucei energy metabolism. RNA 18:1968–1983. doi:10.1261/rna.033829.112 22966087 PMC3479388

[30] Parker F , Maurier F , Delumeau I , Duchesne M , Faucher D , Debussche L , Dugue A , Schweighoffer F , Tocque B . 1996. A Ras-GTPase-activating protein SH3-domain-binding protein. Mol Cell Biol 16:2561–2569. doi:10.1128/MCB.16.6.2561 8649363 PMC231246

[31] Irvine K , Stirling R , Hume D , Kennedy D . 2004. Rasputin, more promiscuous than ever: a review of G3BP. Int J Dev Biol 48:1065–1077. doi:10.1387/ijdb.041893ki 15602692

[32] Sahoo PK , Lee SJ , Jaiswal PB , Alber S , Kar AN , Miller-Randolph S , Taylor EE , Smith T , Singh B , Ho T-Y , Urisman A , Chand S , Pena EA , Burlingame AL , Woolf CJ , Fainzilber M , English AW , Twiss JL . 2018. Axonal G3BP1 stress granule protein limits axonal mRNA translation and nerve regeneration. Nat Commun 9:3358. doi:10.1038/s41467-018-05647-x 30135423 PMC6105716

[33] Lee AK , Klein J , Fon Tacer K , Lord T , Oatley MJ , Oatley JM , Porter SN , Pruett-Miller SM , Tikhonova EB , Karamyshev AL , Wang Y-D , Yang P , Korff A , Kim HJ , Taylor JP , Potts PR . 2020. Translational repression of G3BP in cancer and germ cells suppresses stress granules and enhances stress tolerance. Mol Cell 79:645–659. doi:10.1016/j.molcel.2020.06.037 32692974

[34] Ule J , Jensen KB , Ruggiu M , Mele A , Ule A , Darnell RB . 2003. CLIP identifies Nova-regulated RNA networks in the brain. Science 302:1212–1215. doi:10.1126/science.1090095 14615540

[35] Wang T , Xiao G , Chu Y , Zhang MQ , Corey DR , Xie Y . 2015. Design and bioinformatics analysis of genome-wide CLIP experiments. Nucleic Acids Res 43:5263–5274. doi:10.1093/nar/gkv439 25958398 PMC4477666

[36] Wheeler EC , Van Nostrand EL , Yeo GW . 2018. Advances and challenges in the detection of transcriptome-wide protein-RNA interactions. Wiley Interdiscip Rev RNA 9:e1436. doi:10.1002/wrna.1436 28853213 PMC5739989

[37] König J , Zarnack K , Rot G , Curk T , Kayikci M , Zupan B , Turner DJ , Luscombe NM , Ule J . 2010. iCLIP reveals the function of hnRNP particles in splicing at individual nucleotide resolution. Nat Struct Mol Biol 17:909–915. doi:10.1038/nsmb.1838 20601959 PMC3000544

[38] Krakau S , Richard H , Marsico A . 2017. PureCLIP: capturing target-specific protein-RNA interaction footprints from single-nucleotide CLIP-seq data. Genome Biol 18:240. doi:10.1186/s13059-017-1364-2 29284540 PMC5746957

[39] Maris C , Dominguez C , Allain F-T . 2005. The RNA recognition motif, a plastic RNA-binding platform to regulate post-transcriptional gene expression. FEBS J 272:2118–2131. doi:10.1111/j.1742-4658.2005.04653.x 15853797

[40] Fadda A , Ryten M , Droll D , Rojas F , Färber V , Haanstra JR , Merce C , Bakker BM , Matthews K , Clayton C . 2014. Transcriptome-wide analysis of trypanosome mRNA decay reveals complex degradation kinetics and suggests a role for co-transcriptional degradation in determining mRNA levels. Mol Microbiol 94:307–326. doi:10.1111/mmi.12764 25145465 PMC4285177

[41] Love MI , Huber W , Anders S . 2014. Moderated estimation of fold change and dispersion for RNA-seq data with DESeq2. Genome Biol 15:550. doi:10.1186/s13059-014-0550-8 25516281 PMC4302049

[42] Schmidt EK , Clavarino G , Ceppi M , Pierre P . 2009. SUnSET, a nonradioactive method to monitor protein synthesis. Nat Methods 6:275–277. doi:10.1038/nmeth.1314 19305406

[43] Kullmann M , Göpfert U , Siewe B , Hengst L . 2002. ELAV/Hu proteins inhibit p27 translation via an IRES element in the p27 5′UTR. Genes Dev 16:3087–3099. doi:10.1101/gad.248902 12464637 PMC187493

[44] Mazan-Mamczarz K , Galbán S , López de Silanes I , Martindale JL , Atasoy U , Keene JD , Gorospe M . 2003. RNA-binding protein HuR enhances P53 translation in response to ultraviolet light irradiation. Proc Natl Acad Sci U S A 100:8354–8359. doi:10.1073/pnas.1432104100 12821781 PMC166233

[45] Alexa A , Rahnenführer J , Lengauer T . 2006. Improved scoring of functional groups from gene expression data by decorrelating GO graph structure. Bioinformatics 22:1600–1607. doi:10.1093/bioinformatics/btl140 16606683

[46] Panigrahi AK , Zíková A , Dalley RA , Acestor N , Ogata Y , Anupama A , Myler PJ , Stuart KD . 2008. Mitochondrial complexes in Trypanosoma brucei: a novel complex and a unique oxidoreductase complex. Mol Cell Proteomics 7:534–545. doi:10.1074/mcp.M700430-MCP200 18073385

[47] Mazet M , Morand P , Biran M , Bouyssou G , Courtois P , Daulouède S , Millerioux Y , Franconi J-M , Vincendeau P , Moreau P , Bringaud F . 2013. Revisiting the central metabolism of the bloodstream forms of Trypanosoma brucei: production of acetate in the mitochondrion is essential for parasite viability. PLoS Negl Trop Dis 7:e2587. doi:10.1371/journal.pntd.0002587 24367711 PMC3868518

[48] Creek DJ , Mazet M , Achcar F , Anderson J , Kim D-H , Kamour R , Morand P , Millerioux Y , Biran M , Kerkhoven EJ , Chokkathukalam A , Weidt SK , Burgess KEV , Breitling R , Watson DG , Bringaud F , Barrett MP , Landfear S . 2015. Probing the metabolic network in bloodstream-form Trypanosoma brucei using untargeted metabolomics with stable isotope labelled glucose. PLoS Pathog 11:e1004689. doi:10.1371/journal.ppat.1004689 25775470 PMC4361558

[49] Zíková A , Verner Z , Nenarokova A , Michels PAM , Lukeš J . 2017. A paradigm shift: the mitoproteomes of procyclic and bloodstream Trypanosoma brucei are comparably complex. PLoS Pathog 13:e1006679. doi:10.1371/journal.ppat.1006679 29267392 PMC5739487

[50] Spitznagel D , Ebikeme C , Biran M , Nic a’ Bháird N , Bringaud F , Henehan GTM , Nolan DP . 2009. Alanine aminotransferase of Trypanosoma brucei– a key role in proline metabolism in procyclic life forms. FEBS J 276:7187–7199. doi:10.1111/j.1742-4658.2009.07432.x 19895576

[51] Sykes SE , Hajduk SL . 2013. Dual functions of α-ketoglutarate dehydrogenase E2 in the Krebs cycle and mitochondrial DNA inheritance in Trypanosoma brucei. Eukaryot Cell 12:78–90. doi:10.1128/EC.00269-12 23125353 PMC3535839

[52] Hammond DJ , Gutteridge WE . 1984. Purine and pyrimidine metabolism in the trypanosomatidae. Mol Biochem Parasitol 13:243–261. doi:10.1016/0166-6851(84)90117-8 6396514

[53] Davies MJ , Ross AM , Gutteridge WE . 1983. The enzymes of purine salvage in Trypanosoma cruzi, Trypanosoma brucei and Leishmania mexicana. Parasitology 87:211–217. doi:10.1017/s0031182000052574 6316234

[54] Kovářová J , Barrett MP . 2016. The pentose phosphate pathway in parasitic trypanosomatids. Trends Parasitol 32:622–634. doi:10.1016/j.pt.2016.04.010 27174163

[55] Deschamps-Francoeur G , Simoneau J , Scott MS . 2020. Handling multi-mapped reads in RNA-seq. Comput Struct Biotechnol J 18:1569–1576. doi:10.1016/j.csbj.2020.06.014 32637053 PMC7330433

[56] Melo do Nascimento L , Egler F , Arnold K , Papavasiliou N , Clayton C , Erben E . 2021. Functional insights from a surface antigen mRNA-bound proteome. Elife 10:e68136. doi:10.7554/eLife.68136 33783358 PMC8051951

[57] Alsford S , Turner DJ , Obado SO , Sanchez-Flores A , Glover L , Berriman M , Hertz-Fowler C , Horn D . 2011. High-throughput phenotyping using parallel sequencing of RNA interference targets in the African trypanosome. Genome Res 21:915–924. doi:10.1101/gr.115089.110 21363968 PMC3106324

[58] Shi Z , Barna M . 2015. Translating the genome in time and space: specialized ribosomes, RNA regulons, and RNA-binding proteins. Annu Rev Cell Dev Biol 31:31–54. doi:10.1146/annurev-cellbio-100814-125346 26443190

[59] Nett IRE , Martin DMA , Miranda-Saavedra D , Lamont D , Barber JD , Mehlert A , Ferguson MAJ . 2009. The phosphoproteome of bloodstream form Trypanosoma brucei, causative agent of African sleeping sickness. Mol Cell Proteomics 8:1527–1538. doi:10.1074/mcp.M800556-MCP200 19346560 PMC2716717

[60] Evdokimova V , Ruzanov P , Anglesio MS , Sorokin AV , Ovchinnikov LP , Buckley J , Triche TJ , Sonenberg N , Sorensen PHB . 2006. Akt-mediated YB-1 phosphorylation activates translation of silent mRNA species. Mol Cell Biol 26:277–292. doi:10.1128/MCB.26.1.277-292.2006 16354698 PMC1317623

[61] Matsumoto S , Uchiumi T , Tanamachi H , Saito T , Yagi M , Takazaki S , Kanki T , Kang D . 2012. Ribonucleoprotein Y-box-binding protein-1 regulates mitochondrial oxidative phosphorylation (OXPHOS) protein expression after serum stimulation through binding to OXPHOS mRNA. Biochem J 443:573–584. doi:10.1042/BJ20111728 22280412

[62] Lee C-D , Tu BP . 2015. Glucose-regulated phosphorylation of the PUF protein puf3 regulates the translational fate of its bound mRNAs and association with RNA granules. Cell Rep 11:1638–1650. doi:10.1016/j.celrep.2015.05.014 26051939 PMC4472502

[63] Tyler Weisbarth R , Das A , Castellano P , Fisher MA , Wu H , Bellofatto V . 2018. The Trypanosoma cruzi RNA-binding protein RBP42 is expressed in the cytoplasm throughout the life cycle of the parasite. Parasitol Res 117:1095–1104. doi:10.1007/s00436-018-5787-9 29473141 PMC5866238

[64] Wirtz E , Leal S , Ochatt C , Cross GA . 1999. A tightly regulated inducible expression system for conditional gene knock-outs and dominant-negative genetics in Trypanosoma brucei. Mol Biochem Parasitol 99:89–101. doi:10.1016/s0166-6851(99)00002-x 10215027

[65] Shen S , Arhin GK , Ullu E , Tschudi C . 2001. In vivo epitope tagging of Trypanosoma brucei genes using a one step PCR-based strategy. Mol Biochem Parasitol 113:171–173. doi:10.1016/s0166-6851(00)00383-2 11254965

[66] Das A , Bellofatto V . 2009. The non-canonical CTD of RNAP-II is essential for productive RNA synthesis in Trypanosoma brucei. PLoS One 4:e6959. doi:10.1371/journal.pone.0006959 19742309 PMC2734056

[67] Huppertz I , Attig J , D’Ambrogio A , Easton LE , Sibley CR , Sugimoto Y , Tajnik M , König J , Ule J . 2014. iCLIP: protein-RNA interactions at nucleotide resolution. Methods 65:274–287. doi:10.1016/j.ymeth.2013.10.011 24184352 PMC3988997

[68] Busch A , Brüggemann M , Ebersberger S , Zarnack K . 2020. iCLIP data analysis: a complete pipeline from sequencing reads to RBP binding sites. Methods 178:49–62. doi:10.1016/j.ymeth.2019.11.008 31751605

[69] Roehr JT , Dieterich C , Reinert K . 2017. Flexbar 3.0 - SIMD and multicore parallelization. Bioinformatics 33:2941–2942. doi:10.1093/bioinformatics/btx330 28541403

[70] Smith T , Heger A , Sudbery I . 2017. UMI-tools: modeling sequencing errors in unique molecular Identifiers to improve quantification accuracy. Genome Res 27:491–499. doi:10.1101/gr.209601.116 28100584 PMC5340976

[71] Dobin A , Davis CA , Schlesinger F , Drenkow J , Zaleski C , Jha S , Batut P , Chaisson M , Gingeras TR . 2013. STAR: ultrafast universal RNA-seq aligner. Bioinformatics 29:15–21. doi:10.1093/bioinformatics/bts635 23104886 PMC3530905

[72] Quinlan AR , Hall IM . 2010. BEDTools: a flexible suite of utilities for comparing genomic features. Bioinformatics 26:841–842. doi:10.1093/bioinformatics/btq033 20110278 PMC2832824

[73] Trapnell C , Hendrickson DG , Sauvageau M , Goff L , Rinn JL , Pachter L . 2013. Differential analysis of gene regulation at transcript resolution with RNA-seq. Nat Biotechnol 31:46–53. doi:10.1038/nbt.2450 23222703 PMC3869392

[74] Ross PL , Huang YN , Marchese JN , Williamson B , Parker K , Hattan S , Khainovski N , Pillai S , Dey S , Daniels S , Purkayastha S , Juhasz P , Martin S , Bartlet-Jones M , He F , Jacobson A , Pappin DJ . 2004. Multiplexed protein quantitation in Saccharomyces cerevisiae using amine-reactive Isobaric tagging reagents. Mol Cell Proteomics 3:1154–1169. doi:10.1074/mcp.M400129-MCP200 15385600

[75] Su X , Chiles E , Maimouni S , Wondisford FE , Zong W-X , Song C . 2020. In-source CID ramping and covariant ion analysis of hydrophilic interaction chromatography metabolomics. Anal Chem 92:4829–4837. doi:10.1021/acs.analchem.9b04181 32125145 PMC8141260

[76] Supek F , Bošnjak M , Škunca N , Šmuc T . 2011. REVIGO summarizes and visualizes long lists of gene ontology terms. PLoS One 6:e21800. doi:10.1371/journal.pone.0021800 21789182 PMC3138752

[77] Luo W , Friedman MS , Shedden K , Hankenson KD , Woolf PJ . 2009. GAGE: generally applicable gene set enrichment for pathway analysis. BMC Bioinformatics 10:161. doi:10.1186/1471-2105-10-161 19473525 PMC2696452

[78] Luo W , Brouwer C . 2013. Pathview: an R/Bioconductor package for pathway-based data integration and visualization. Bioinformatics 29:1830–1831. doi:10.1093/bioinformatics/btt285 23740750 PMC3702256

[79] Kanehisa M , Goto S . 2000. KEGG: kyoto encyclopedia of genes and genomes. Nucleic Acids Res 28:27–30. doi:10.1093/nar/28.1.27 10592173 PMC102409

